# Whole Genome Microarray Analysis of DUSP4-Deletion Reveals A Novel Role for MAP Kinase Phosphatase-2 (MKP-2) in Macrophage Gene Expression and Function

**DOI:** 10.3390/ijms20143434

**Published:** 2019-07-12

**Authors:** Thikryat Neamatallah, Shilan Jabbar, Rothwelle Tate, Juliane Schroeder, Muhannad Shweash, James Alexander, Robin Plevin

**Affiliations:** 1 Department of Pharmacology and Toxicology, Faculty of Pharmacy, King Abdulaziz University, P.O. Box 80260, Jeddah 21589, Saudi Arabia; 2 Strathclyde Institute for Pharmacy & Biomedical Sciences, University of Strathclyde, 161 Cathedral Street, Glasgow G4 0RE, UK

**Keywords:** MAP Kinase Phosphatase-2, DUSP-4, macrophages, proliferation, differentiation

## Abstract

Background: Mitogen-activated protein kinase phosphatase-2 (MKP-2) is a type 1 nuclear dual specific phosphatase (DUSP-4). It plays an important role in macrophage inflammatory responses through the negative regulation of Mitogen activated protein kinase (MAPK) signalling. However, information on the effect of MKP-2 on other aspect of macrophage function is limited. Methods: We investigated the impact of MKP-2 in the regulation of several genes that are involved in function while using comparative whole genome microarray analysis in macrophages from MKP-2 wild type (wt) and knock out (ko) mice. Results: Our data showed that the lack of MKP-2 caused a significant down-regulation of colony-stimulating factor-2 (*Csf2*) and monocyte to macrophage-associated differentiation (*Mmd*) genes, suggesting a role of MKP-2 in macrophage development. When treated with macrophage colony stimulating factor (M-CSF), *Mmd* and *Csf2* mRNA levels increased but significantly reduced in ko cells in comparison to wt counterparts. This effect of MKP-2 deletion on macrophage function was also observed by cell counting and DNA measurements. On the signalling level, M-CSF stimulation induced extracellular signal-regulated kinases (ERK) phosphorylation, which was significantly enhanced in the absence of MKP-2. Pharmacological inhibition of ERK reduced both *Csf2* and *Mmd* genes in both wild type and ko cultures, which suggested that enhanced ERK activation in ko cultures may not explain effects on gene expression. Interestingly other functional markers were also shown to be reduced in ko macrophages in comparison to wt mice; the expression of CD115, which is a receptor for M-CSF, and CD34, a stem/progenitor cell marker, suggesting global regulation of gene expression by MKP-2. Conclusions: Transcriptome profiling reveals that MKP-2 regulates macrophage development showing candidate targets from monocyte-to-macrophage differentiation and macrophage proliferation. However, it is unclear whether effects upon ERK signalling are able to explain the effects of DUSP-4 deletion on macrophage function.

## 1. Introduction

It is now well recognized that the mitogen-activated protein (MAP) kinases are the essential regulators of a diverse range of immune responses that are linked to normal physiology and disease [1]. The MAP kinase phosphatases (MKPs) finely regulate MAP kinase activity in cells [2]. This group consists of a ten membered family of dual specific phosphatases (DUSPs) that function to terminate MAP kinase signaling within a defined subcellular location. There are three classes I, II, and III defined by subcellular location, specificity for each MAP kinase and the modes of regulation [3]. For example, MKP-1 is a nuclear DUSP of the type 1 class and is selective for all three major MAP kinases in vitro, whilst MKP-3, which is a type II DUSP, is a cytosolic phosphatase selective solely for ERK over the other kinases. 

Previous evidence indicates the potential of DUSPs to play an important role in the regulation of immune function [4]. It has been shown that the deletion of MKP-1 results in severe exacerbation of septic shock and arthritis via enhanced activity of p38 and JNK [5,6]. In contrast, PAC1 (DUSP-2) deletion mice have reduced inflammatory responses in arthritis [7], whilst the deletion of MKP-5 (DUSP-10) results in marked changes in T- cell proliferation and in CD4^+^ and CD8^+^ function [8]. 

MAP Kinase phosphatase-2 (MKP-2) is a type 1 DUSP that is nuclear located due to the expression of two nuclear localization sequences (NLS) [9]. MKP-2 is selective for ERK and JNK in vitro [10] and is induced by multiple agents, including serum, growth factors, UV-light, and oxidative stress (see review by [11]). In comparison to other DUSPs, in particular, MKP-1, the function of MKP-2 has not been as comprehensively studied and its role within the immune system has not been well defined. Using a novel DUSP-4 deletion mouse model we have demonstrated a key role for MKP-2 in regulating infection mediated by either *Leishmania mexicana* [12], *Leishmania donovani* [13], or *Toxoplasma gondii* [14]. The responses are underpinned by an enhanced macrophage expression of Arginase-1 and reduced expression of iNOS in two of these models [12]. Other studies have implicated a role for DUSP-4 in mediating sepsis via the regulation of cytokine production [15] and in T- cell proliferation [16]. However, the exact role of MKP-2 in regulating macrophage gene expression and function is not fully investigated.

We conducted a comparative microarray gene expression analysis on MKP-2 wt and ko macrophages following lipopolysaccharide (LPS) activation in this study. We examined the impact of MKP-2 in the regulation of several genes that are involved in macrophage development and function for the first time. In particular, we identified a role for MKP-2 in the expression of two genes that are related to macrophage development, *Csf2,* and *Mmd*. We further confirmed the correlation of both genes and the defect in macrophage development linked to MKP-2 loss. Additionally, we further confirmed the effect of MKP-2 deletion on the expression of progenitor markers CD115 and CD34, suggesting a more global effect for DUSP-4 in macrophage function.

## 2. Results

### 2.1. The Effect of MKP-2 in the Gene Expression Pattern of LPS-Stimulated Macrophages

The relative importance of MKP-2 in macrophage function was studied using microarray gene expression analysis. A four-hour LPS stimulation was selected to cover early and late innate immune response genes in macrophages since most of the genes are induced or repressed in a temporal pattern. Generally, LPS induced the expression of a large number of cytokines, chemokines, and growth factors with disparate expressions between wt and MKP-2-ko macrophages. Table 1 shows the top 10-upregulated Agilent SurePrint G3 (8× 60K) array probes in response to LPS. Gene ontology analysis showed that highly enriched biological processes following LPS stimulation in both MKP-2-wt and ko macrophages are related to immune function (Table 2). Genes, such as *Il19, Cxcl1, Cxcl2 Prst1,* and *Il1a,* were more enhanced in MKP-2^+/+^ when compared to MKP-2^-/-^ counterparts. However, a slight increase was recorded for *Il1b*, *Ifng*, *Cxcl10*, and *Ccl22* in MKP-2-deficient macrophages in relation to wt cells. In addition, the gene encoding CD14 was also upregulated in MKP-2^+/+^ over MKP-2^-/-^ macrophages (27.9 vs. 10.1, respectively). CD14 is a surface antigen that is expressed on monocytes and macrophages to mediate the innate immune response to bacterial lipopolysaccharide). The decrease in *CD14* upregulation by MKP-2^-/-^ macrophages could explain the attenuation in the inflammatory response in these macrophages, as CD14 is critical for the recognition of LPS by macrophages to initiate the innate immunity and release of cytokines and chemokines.

Among the highly induced genes, *Gfi1* was also differently expressed in MKP-2 wt versus ko LPS-induced macrophages (1574.1 vs. 663.9, respectively). Gfi-1 is a transcriptional repressor that regulates the Toll-like receptor (TLR) inflammatory response by antagonising the NF-κB pathway [17]. Endothelin-1 (*Edn1),* a gene that is encoded for a vasoconstrictor protein EDN-1, significantly differed between LPS-stimulated MKP-2 wt and ko macrophages (1094 vs. 5176, respectively, *p*-value < 0.05), with *Edn1* showing the highest fold change of any gene in the induced MKP-2 ko group.

**Figure 1:**
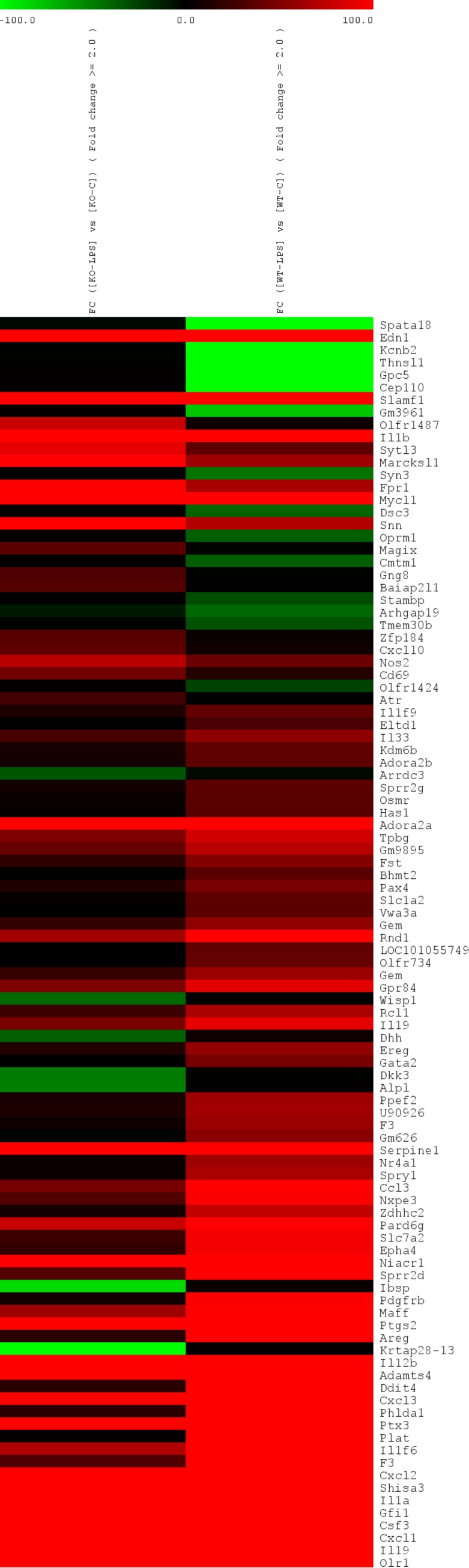
Heat maps representing hierarchical clustering of the differentially regulation, plotted using the log2 fold change (FC) values of the genes with *p*-value ≤0.05 (unpaired *t*-test) between Mitogen-activated protein kinase phosphatase-2 (MKP-2)^+/+^ vs. MKP-2^-/-^. Each column represents a sample and each row in the heat map represents a gene that is differentially regulated in that particular comparison of samples. Signal intensities were normalized to the expression data of unstimulated macrophages. The color scale represents the degree of expression of the gene, green being the downregulated (below −100) and red being (above +100) the upregulated genes in the sample sets with black as the center of the scale at ‘0′.

Interestingly, genes for macrophage development, such as monocyte to macrophage differentiation associated (*Mmd*) and colony-stimulating factor-2 (*Csf2*), were also upregulated following LPS stimulation (FC= 33.7- and 24.5- for wt, respectively, vs. 17.6 and 7.8 for ko, respectively. *Csf2* encodes for CSF-2 cytokine or GM-CSF that controls the production, differentiation and growth of granulocytes and macrophages [18]. *Mmd* is mainly upregulated upon monocyte differentiation and it is expressed in mature, in vitro differentiated macrophages whilst missing in monocytes [19]. Figure 1 illustrates the expression differences of significantly regulated genes in the LPS-stimulated macrophages.

### 2.2. The Role of MKP-2 in the Expression of M-CSF-Induced Macrophage Differentiation and Proliferation Genes

**Figure 2:**
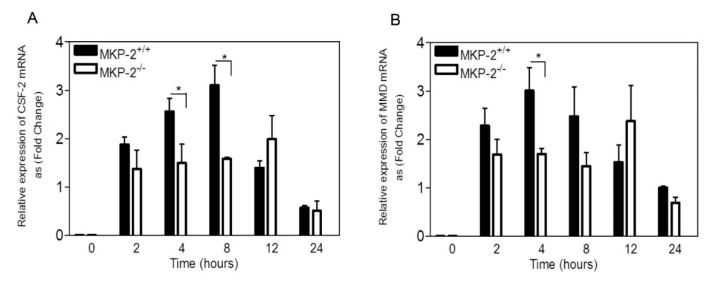
MKP-2 deletion negatively affects *Csf2* and *Mmd* gene expression in bone marrow-derived macrophages. Cells from day 3 of culture, were rendered quiescent in macrophage colony stimulating factor (M-CSF) free medium and then stimulated with recombinant M-CSF (10 ng/ml) for the indicated periods of time. Control cells (0) were left untreated. Total RNA was prepared from the cells. After reverse transcription, quantitative PCR analysis was performed on cDNA while using primers designed to detect *Csf2* (**A**) and *Mmd* (**B**). Expression levels of *Mmd* and *Csf-2* mRNA transcripts were normalized to the reference gene *QARS* using the delta-delta Ct method [20]. Error bars represent the mean ± SEM from three individual experiments. * *p* < 0.05, one-tailed unpaired t test comparing MKP-2^-/-^ to MKP-2^+/+^ macrophages.

Having identified the differential expression of macrophage genes following LPS stimulation, changes in *Edn1* mRNA, and protein levels were further confirmed (Supplementary Figure S1). This validated, while using RT-qPCR, the microarray data as a bonafide measure of gene expression. Thus, using RT-qPCR, we then examined the differential expression of important macrophage development genes, *Csf2* and *Mmd.* Macrophage colony stimulating factor (M-CSF), which is a specific growth factor for macrophages development, was used to stimulate the cells. As shown in Figure 2A, the induction of *Csf2* in MKP-2^+/+^ macrophages by M-CSF was increased at 2 h, reaching a peak by 8 h, and decreased again by 24 h (fold stimulation at 8 h = 2.45 ± 0.85). Surprisingly, in MKP-2^-/-^ macrophages, the expression of *Csf2* was reduced between 2 and 8 h, reaching a significant difference at 4 and 8 h (*p* < 0.05). However, the level of *Csf2* was slightly but not significantly enhanced in MKP-2^-/-^ macrophages at 12 h (Figure 2A). During the same time course and, as demonstrated in Figure 2B, *Mmd* mRNA levels were increased in MKP-2^+/+^ macrophages at 2 h, reaching a peak by 4 h and decreased by 24 h (fold stimulation at 4 h = 2.53 ± 0.5). Similar to *Csf2, Mmd* expression in MKP-2^-/-^ macrophages was reduced at 2, 4, and 8h, reaching a significant difference at 4 h (*p* < 0.05). This demonstrates that MKP-2 can be an important factor in the maximal regulation of *Mmd* and *Csf2* gene expression in the early stages of stimulation. The increase of *Mmd* and *Csf2* mRNA at 12 h in MKP-2^-/-^mice suggests a late response from this group to induce both genes (Figure 2A,B).

### 2.3. MKP-2 Deletion Negatively Effects M-CSF Stimulated Macrophage Proliferation

**Figure 3:**
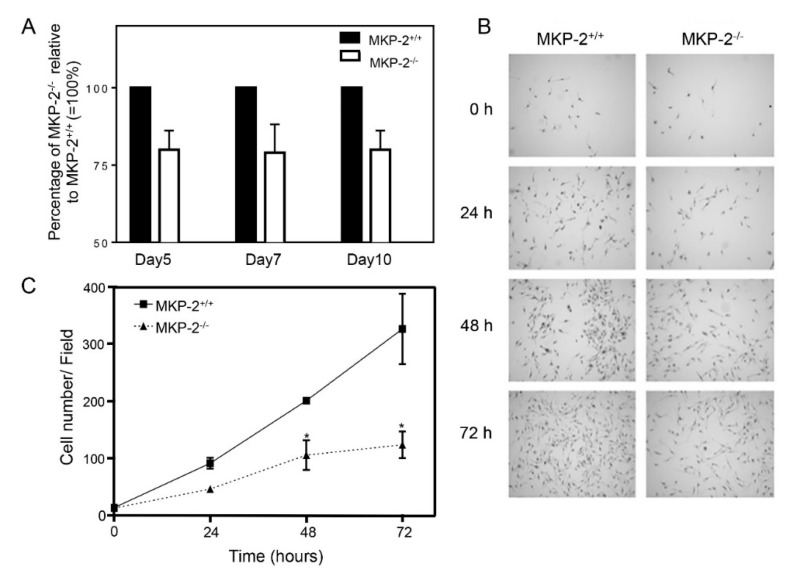
MKP-2 deletion reduces proliferative of bone marrow macrophages. (**A**) An equal number of cells (3.5 × 10^6^) from the bone marrow cell suspension were cultured for 10 days in media supplemented with 30% L-929 conditioned medium (LCM) as a source for M-CSF. Macrophages were harvested from petri dishes at days 5, 7, and 10 following isolation. (**B**,**C**) Cells from day 3 of culture were rendered quiescent in cover slips for 24 h in M-CSF free medium and then stimulated with recombinant M-CSF (10 ng/mL) for the times indicated or left unstimulated (Controls, 0 h). The number of cells was determined, as described in Section 4.7.1. Control values, (0 h) MKP-2^+/+^ = 20, MKP-2^-/-^ = 18. Each value represents the mean ± SEM from three individual experiments. * *p* < 0.05, two-tailed unpaired *t*-test comparing MKP-2^-/-^ to MKP-2^+/+^ macrophages. Images were taken using a 20× objective, scale bar = 50 µm.

**Figure 4:**
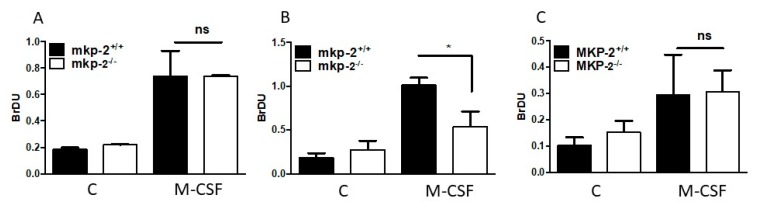
Effect of MKP-2 deletion on macrophage proliferation. Macrophages from both MKP-2^+/+^ and MKP-2^-/-^ were harvested on day 3 of culture, seeded at 4 × 10^4^ cells/well in a 96-well plate and incubated overnight. Cells were stimulated then with 10 ng/ml M-CSF for (**A**) 24 h, (**B**) 48, and (**C**) 72 h. 10µM BrDU was added to each well. The cells were incubated for 6 h. Incorporated BrDU was measured at absorbance of 450 nM. Data represents mean ± SEM of three independent experiments. * *p* < 0.05 two-tailed unpaired *t*-test comparing MKP-2^-/-^ to MKP-2^+/+^ macrophages.

The proliferative potential of macrophages following MKP-2 deletion was examined over a period of ten days using L929-conditioned medium, having established the negative regulation of *Csf2* and *Mmd* genes following MKP-2 deletion. Macrophages were harvested on days 5, 7, and 10 and counted in a haemocytometer. In the absence of MKP-2, macrophage proliferation was reduced by 20.04%, 21.1%, and 20.1% at days 5, 7, and 10, respectively, in comparison to wild type cells (Figure 3A). We assessed the proliferative capacity of macrophages over 72 h to further confirm our data. In MKP-2^+/+^ macrophages, M-CSF was able to increase the number of MKP-2^+/+^ cells 13-fold over 72 h (Figure 3B,C). In contrast, M-CSF-stimulated proliferation was significantly slower at 48 and 72 h when compared to wild type (*p* < 0.05) in MKP-2^-/-^ macrophages. Next, we studied macrophage proliferation and confirmed itusing a BrdU cell proliferation assay. Cells from both MKP-2^+/+^ and MKP-2^-/-^ mice were harvested on day three and the proliferative rate was tracked for up to 72 h, which would be day seven, the time point when the macrophages are expected to be fully differentiated. The results in Figure 4A indicate a significant increase in the macrophage proliferation rate in both MKP-2^+/+^ and MKP-2^-/-^ after 24 h of M-CSF stimulation. This was approximately 3–4 fold in both populations with a slight increase in the wild type MKP-2 population over MKP-2 deleted macrophages. After 48 h (Figure 4B), proliferation further increased in the wt MKP-2 macrophages, however in MKP-2 ko macrophages, cell growth significantly lagged. By 72 h, the proliferation rates for both cultures had fallen and they were similar (Figure 4C). This provided additional evidence that the deletion of MKP-2 significantly impairs the growth of macrophages from precursors and that MKP-2 is essential for appropriate macrophage proliferation.

### 2.4. MKP-2 Deletion Enhances ERK Kinase Signaling

**Figure 5:**
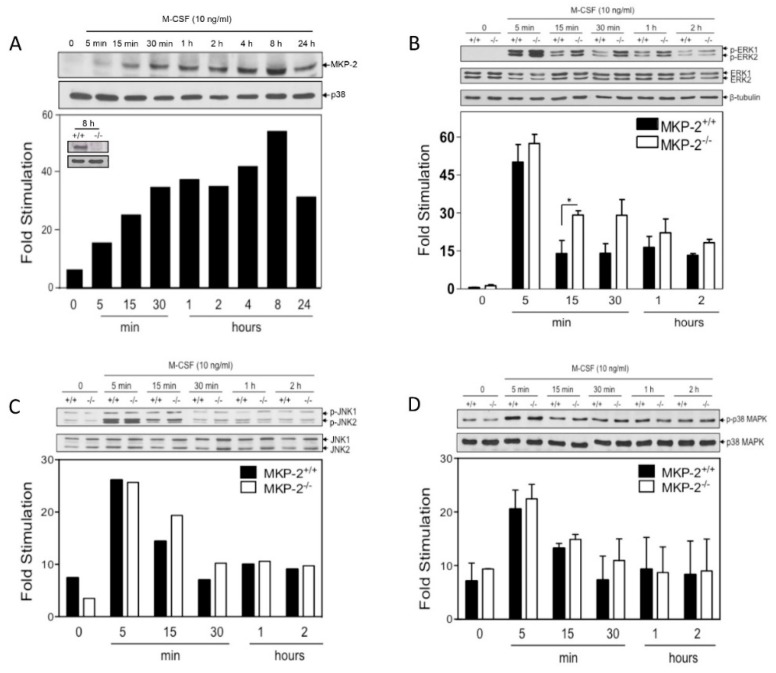
MKP-2 deletion enhances M-CSF-mediated ERK phosphorylation in macrophages. Cells harvested on day 3 of culture, were rendered quiescent in M-CSF free medium then stimulated with recombinant M-CSF (10 ng/mL) for the times indicated. Control cells (0) were left untreated. Whole cell lysates were prepared, and assessed for (**A**) MKP-2 (43 kDa) (**B**) p-ERK (42/44 kDa), (**C**) p-JNK (46/65 kDa), and (**D**) p-p38 (38 kDa) or total controls by Western blotting, as described in the Methods. MKP-2 was not induced in cells that were derived from MKP-2^−/−^ mice (upper left insert of Panel A). Blots were semi-quantified for fold stimulation by scanning densitometry relative to the background signal. The blot represents the data from 2–3 individual experiments. * *p* < 0.05, one-tailed unpaired *t* test comparing MKP-2^-/-^ to MKP-2^+/+^ macrophages.

We studied the relationship between MKP-2 expression, MAP kinase signaling, and whether MAPK activation was necessary for the proliferative response of macrophages to M-CSF. M-CSF stimulated a rapid increase in MKP-2 expression, which was detected as early as 15 minutes, and was maximal, by 1 h (Figure 5A). No MKP-2 expression was observed in equivalently stimulated MKP-2^-/-^ macrophages (see Figure 5A insert).

In the absence of MKP-2, ERK phosphorylation by M-CSF was enhanced relative to wild type controls (Figure 5B). Studying a time course between 5 to 60 minutes revealed a significant difference at 15 min. (fold stimulation at 15 minutes: MKP-2^+/+^ = 16.26 ± 8.04, MKP-2^-/-^ = 29.59 ± 2.82, *p* < 0.05). This response declined at 2 h in both genotypes. In contrast, there were no measurable differences in the phosphorylation of JNK and p38 MAPK between MKP-2^+/+^ and MKP-2^-/-^ macrophages over the same time course (Figure 5C,D). These data indicate that M-CSF-induced ERK activation was selectively enhanced in MKP-2 deficient macrophages in comparison to wild type.

### 2.5. The Effect of ERK Inhibition on M-CSF Induced Proliferation and Mmd and Csf2 Expression

**Figure 6:**
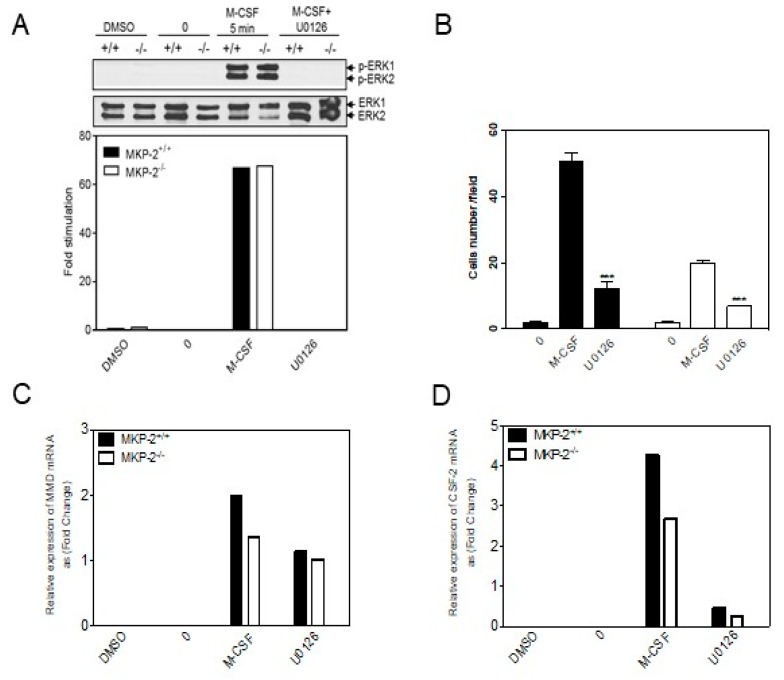
The effect of MAPK inhibition on M-CSF induced MAPK phosphorylation and *Csf2/Mmd* expression. (**A**) Cells from day 3 of culture were rendered quiescent in M-CSF free medium and then pre-incubated with DMSO (vehicle control) or 10 µM of U0126 for 1 h where indicated. The cells were either left unstimulated (0), or stimulated with M-CSF (10 ng/mL) for 5 min. Whole cell lysates were prepared, and assessed for p-ERK (42/44 kDa) by Western blotting. The blot represents the data from two individual experiments. (**B**) Cells that were harvested on day 3 of culture (5 × 10^3^) were rendered quiescent in coverslips for 24 h in M-CSF free medium and then pre-treated with (U0126) for 1 h prior to stimulation with recombinant M-CSF (10 ng/ml) for 48 h, stimulated with M-CSF alone or left unstimulated (0 h). Each value represents the mean ± SEM from three individual experiments. *** *p* < 0.001, one-tailed unpaired t test relative to 48 h M-CSF treated macrophages. (**C**,**D**) Relative expression levels of *Mmd* mRNA transcripts were normalized to the reference gene *QARS* while using the delta-delta Ct method [17]. The figure represents the data from three individual experiments.

Differences in the activation of ERK following MKP-2 deletion suggest the potential involvement of ERK activation in macrophage proliferation. The effect of ERK inhibition on M-CSF-induced ERK phosphorylation was examined to study this correlation. The results that are presented in Figure 6A show that the pre-treatment of cells with U0126 completely inhibited ERK phosphorylation at 5 min in both genotypes relative to their respective control.

An additional ERK inhibition experiment was carried out to investigate the definitive role of ERK in macrophage proliferation, since MKP-2 deletion was found to negatively affect proliferation (Figure 3). This was achieved by studying the effect of MEK1,2 inhibitor, U0126, on macrophage proliferation using cell counting using hematoxylin staining. M-CSF was generally able to increase the number of cells at 48 h, but to a greater extent in wild type cultures (Figure 6B), a finding that is consistent with previous experiments. Inhibiting ERK activity while using U0126 significantly impairedcell growth, but to an equivalent extent in each genotype (Figure 6B). This experiment provided additional evidence that ERK significantly regulates the proliferation of bone marrow derived macrophages from precursors and that ERK activation by M-CSF is essential for appropriate macrophage development.

The effect of ERK inhibition on *Mmd* and *Csf2* expression was also examined (Figure 6C,D). The results presented in Figure 6C show that, relative to stimulated controls, the pre-treatment of cells with U0126 reduced M-CSF-induced *Mmd* expression by ~52.2% and ~33% for wild type and knock-out cells, respectively (Figure 6C). In contrast, ERK inhibition substantially reduced the *Csf2* mRNA levels in both wild type and knock-out cells, where inhibition was approximately 85% for each condition. (Figure 6D). This shows for the first time that the expression of both genes is positively regulated through ERK activation. However, taking Figure 5; Figure 6 together, the data show that enhanced ERK signaling in MKP-2 deficient macrophages does not translate into enhanced *Mmd* or *Csf2* gene expression nor increased proliferation, but a reduction in all three parameters.

### 2.6. Differential Expression of CD34 and CD115 on the Surface of Bone Marrow Cells

We next examined the effect of MKP-2 loss on the expression of macrophage surface proteins CD115 and CD34. CD34 is an important progenitor cell marker [21], whereas (CD115) is a receptor for M-CSF that has been used to identify cells that belong to the mononuclear phagocyte system, including macrophages [19].

**Figure 7:**
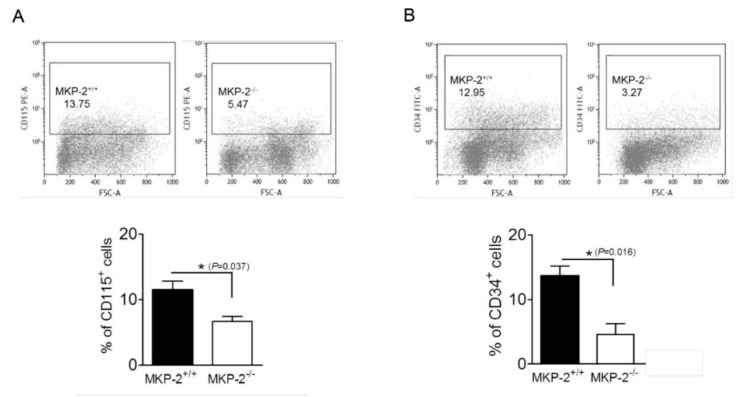
MKP-2 deletion negatively affects CD115 and CD34 expression. Native bone marrow cells were isolated and subsequently analyzed for CD115 and CD34, as described in Section 4.6. The gates represent the CD115 (**A**) or CD34 (**B**) positive population as percent within the native cell suspension. Gating was performed according to negative isotype-matched controls. Flow cytometry data represent the mean ± SEM of three separate experiments. * *p* < 0.05, two-tailed unpaired *t* test comparing MKP-2^-/-^ to MKP-2^+/+^ macrophages.

The analysis of bone marrow cells showed a significant reduction in CD115 expression on cells from MKP-2^-/-^ mice in comparison to wild type; 5.6–8.2% and 9.1–13.8%, respectively, *p* < 0.05 (Figure 7A). Based on this, the bone marrow cell suspension from MKP-2^-/-^ mice contained less macrophage precursors within the population. Analysis of the cell suspension showed that approximately 11.3–16.5% and 2.3–7.8% of the cells were CD34^+^ for MKP-2^+/+^ and MKP-2^-/-^, respectively. The percentage of CD34 positive cells was significantly lower in MKP-2^-/-^ when compared to the wild type counterparts (*p* < 0.05) (Figure 7B). This indicated that MKP-2^-/-^ bone marrow consisted of fewer myeloid progenitors. It is noteworthy that the percentage of CD34^+^CD115^+^ cells was also reduced in MKP-2^-/-^ bone marrow with 6.3%±0.9 when compared to 3.75 ± 0.45 in MKP-2^+/+^ bone marrow.

## 3. Discussion

For the first time we conducted a whole genome array to investigate the role of MKP-2 in the macrophage function, building on the initial finding using a DUSP-4 ko mouse model that showed effects upon inflammatory gene expression and resistance to leishmania and toxoplasma infection [12,14]. The initial results are consistent with previously published studies demonstrating LPS-induced gene programs in murine macrophages [22,23]. We and others have also shown that MKP-2 deletion has an impact on number of cytokines, such as IL-6, IL-12, TNFα, and COX-2 in LPS-stimulated macrophages [12], and these findings were replicated in the whole genome study. However, differences in other genes were also observed, such as CD14 and End1, and in preliminary RT-qPCR experiments, we used *End1* to validate the gene array recapitulating the results at the mRNA and protein level. Endothelin-1 has the potential to regulate the macrophage function [24,25,26], but more importantly smooth muscle contractility and proliferation [27,28], and this is currently being studied.

However, the study also showed that MKP-2 deletion reduced the expression of *Mmd* and *Csf2* genes, both of which are believed to be critical in macrophage differentiation and proliferation. We further showed, for the first time, that these two key genes, *Csf2* and *Mmd* were substantially reduced during the initial phase of stimulation in MKP-2^-/-^ macrophages relative to wild type macrophages following stimulation with M-CSF, which is a key driver of macrophage proliferation and differentiation. The Monocyte to Macrophage Differentiation (*Mmd*) gene was first identified in 1995 and it is up-regulated during the differentiation of monocytes to macrophages [29]. The protein that is encoded by the *Csf2* is a cytokine that controls the production, differentiation, and function of macrophages [18]. Studies examining the regulation and function of these genes are limited, however Liu, Q., et al. has shown that LPS significantly up-regulated *Mmd* expression in macrophages and Mmdover-expression enhanced LPS-stimulated production of TNF-α and NO via ERK and Akt phosphorylation [30]. Similarly, an early study showed that, total cell numbers of blastocysts from CSF-2^−/−^ mice were reduced to 18% compared with wild-type controls [31].

Thus, MKP-2 deletion adversely affected the ability of cells to differentiate into adult macrophages over the 10-day period. Our data also showed a significant reduction in macrophage proliferation in response to M-CSF following to loss of MKP-2. This phenomenon is consistent with studies in fibroblasts that showed a potential effect on G2/M-Phase transition [32]. In those studies, serum or PDGF was used as a stimulant suggesting the involvement of pathways, such as ERK or PI3K/PKB. In this study, we utilized M-CSF [33,34], which interacts with colony stimulating factor-1 receptor (CSFR1) and couples to MAPKs and PI3K/Akt [35,36]. In response to M-CSF, we consistently found that the deletion of MKP-2 enhanced ERK activation, but it had no significant effect upon JNK and p38 activation. This profile is different to that observed in adult macrophages that were stimulated with LPS in which JNK and p38 MAP kinase phosphorylation was increased but ERK activation was not affected [12]. In another MKP-2 deletion model, an increase in ERK phosphorylation in response to LPS has been demonstrated [15]. Significantly, we found that M-CSF stimulated a very rapid expression of MKP-2 relative to other studies, including our own, however it is recognized that ERK activation controls MKP-2 expression [32]. Thus, the extremely rapid activation of ERK by M-CSF would coincide with this profile. It should also be noted that ERK-mediated phosphorylation is also able to promote the stabilization of MKP-2 [37] and this may contribute to the rapid cellular expression in the cells that were stimulated by M-CSF.

A key issue is determining whether enhanced ERK signaling following MKP-2 deletion plays a role in the functional effects that were observed following MKP-2 deletion. To this end, we treated cells with the MEK1 inhibitor U0126. For proliferation, it was found that ERK inhibition significantly reduced the proliferation in both set of cultures. This is consistent with a number of studies that show that ERK is important in M-CSF-dependent macrophage proliferation [38,39] and is able to regulate a number of early genes and is essential for cell cycle progression [40]. This might suggest that the effect of MKP-2 deletion to enhance ERK is not related to the overall negative effect on proliferation. However, several studies have indicated that the kinetics of ERK phosphorylation is significant in determining whether macrophages are stimulated to proliferate or activated immunologically; a rapid transient signal mediates proliferation, a more prolonged signal mediates activation [38,41,42], thus MKP-2 deletion may be steering macrophages towards activation. Consistent with our results, it has been shown that the knock down of MKP-1 in macrophages also leads to prolonged ERK activation and the inhibition of proliferation [43]. A similar argument may be posited for the consistent and inhibition of both *Mmd* and *Csf2* expression following pretreatment with U0126, which again we show for the first time.

However, it is still very possible that the enhancement of ERK signaling is not linked to the negative effect of MKP-2 on the proliferation and expression of *Mmd* and *Csf2*, despite the arguments above. Thus, another potential mechanism might be involved. Recently, Joeng et al. has demonstrated the direct binding of MKP-2 to VRK1, an effect that is independent of phosphatase activity [44]. Given that MKP-2 is nuclear located, it has the potential to positively regulate *Mmd* and *Csf2* gene transcription potential via the effect on Histone H3. However, we also show, for the first time, that a number of key macrophage markers are reduced following DUSP-4 deletion. This includes the expression of the M-CSF receptor, CD115 [45,46], and this finding would explain the consistent inhibitory effect of DUSP-4 deletion on the proliferation and differentiation of macrophages in response to M-CSF. However, we also show that the hematopoietic stem cell progenitors are also significantly reduced. CD34 is an important stem/progenitor cell marker [21] and CD34^+^ myeloid progenitors differentiate into monocytes and further to macrophages in the presence of M-CSF [47]. Thus, macrophages may lack responsiveness due to a number of factors as a result of MKP-2 deficiency but not directly related to kinase activation in the early stages of development. Interestingly, we found that adult macrophage markers where not altered including F4/80, CD14, CD11b and MHC-II (results not shown), thus the deletion is very specific to the early stages of the differentiation process. This may be related to a reduced of progenitor cells to initiate the differentiation with and less receptors responding to M-CSF. Therefore, proliferation will be correspondingly reduced.

A future analysis would therefore help to uncover the potential role of MKP-2 in the early stages of myeloid differentiation. Macrophages are derived from myeloid progenitor cells differentiated from hematopoietic stem cells (HSCs) in the bone marrow [48]. Studies have identified common CD115^+^ CD135^+^ CX3CR1^+^ markers that are associated with the myeloid stem cells committed toward macrophage/DC lineage [49,50]. These and other myeloid precursors generating cells such as neutrophils, eosinophils, basophils and mast cells would have to be analysed. Furthermore, it would be important to determine whether the effect of DUSP4 deletion can generally attenuate other lineages originate from HSCs, such as lymphoid progenitors (LPs), the source of CD19/CD45 B cells and CD4/CD8 T cells. We have already shown the reduced frequency of CD4^+^ and CD8^+^ T cells in spleens upon MKP-2 deletion, which suggests that this lineage is also compromised [51]. However, if DUSP-4 deletion is not affecting the number macrophage progenitors, the function of these cells, including the potential to differentiate, would have to be analysed across a host of cellular parameters, including the expression of differentiation markers, proliferation, and the effect of ERK signaling. A recent study has shown that MAP kinase is linked to macrophage progenitor development [39].

In conclusion, using a whole genome microarray of macrophages from wt and DUSP-4 deletion mice for the first time, we uncover the potential for MKP-2 to regulate a significant number of genes. Two of these, *Mmd* and *Csf-2,* are implicated in the proliferation of macrophages. Furthermore, whilst MKP-2 deletion enhanced ERK signaling, this did not readily explain the effect upon macrophage function, which is profound. Finally, it highlights the importance to carry out further studies on the role of MKP-2 in macrophages and other myeloid cells.

## 4. Materials and Methods

### 4.1. Reagents

The antibodies were purchased as follows: Rabbit polyclonal anti-JNK-1 (S-18), anti-ERK-1 (K-23) and anti-p38 (N-20) antibodies were all purchased from Santa Cruz (Dallas, TX, USA). Anti-phospho-ERK (T202/Y204) and anti-phospho-JNK (T183/Y185) were purchased from Cell Signaling Technology (Hitchin, UK). Anti-phospho-p38 (44684-G) was purchased from Applied Biosystems, Life Technologies (Paisley, UK). HRP- conjugated anti-mouse, and conjugated anti-rabbit antibodies were purchased from Jackson Immunoresearch laboratories (Luton, Beds, UK). Anti-Mouse CD34-FITC conjugated, anti-Mouse CD115 (c-fms)-PE conjugated, and recombinant M-CSF were all purchased from eBioscience (Hatfield, UK). SP600125 JNK inhibitor, SB 203580 p38 inhibitor, and U2106 MEK1,2 inhibitor was purchased from Tocris Bioscience (Bristol, UK). BrdU Cell Proliferation kit was purchased from Cell Signaling (New England Biolabs, Hitchin, UK). SuperScript^®^ III First-Strand Kit (cDNA synthesis system for RT-PCR), oligonucleotide PCR Primers, SYBR^®^ Select real-time PCR Master Mix were purchased from Applied Biosystems, Life Technologies (Paisley, UK). RNeasy Mini Kit (RNA extraction kit), and RNase-Free DNase Set were obtained from QIAGEN (Manchester, UK). All other materials used were of the highest commercial grade available and they were obtained from Sigma Aldrich (Poole, Dorset, UK).

### 4.2. DUSP-4 Deletion Mice

The DUSP-4 deletion mice were generated in collaboration with Geno-way, France, as described previously [12].

### 4.3. Cell Culture

Bone marrow derived macrophages were isolated from femurs and tibia of 6–8 weeks old MKP-2^−/−^ or ^+/+^ mice and that were grown in DMEM (Sigma-Aldrich), containing 10% (*v*/*v*) heat-inactivated FCS (Gibco Life Technologies, paisley) and supplemented with 30% L929 cell-conditioned medium as a source of M-CSF supplemented with 5 mM l-Glutamine, 100 U/mL penicillin, 1 µg/mL streptomycin. After culture, the adherent cells were harvested and then seeded in either 12 well (1 × 10^6^ cells/mL) or 6 well (2 × 10^6^ cells/2 mL) plates with RPMI 1641 supplemented with 10% FCS. The cells at this stage were deprived of M-CSF for 18 h and then treated with M-CSF.

### 4.4. Microarray Expression Analysis

Total RNA was extracted with the RNase Mini Kit, following the manufacturer’s recommendations (QIAGEN, Manchester, UK). RNA concentrations and A260/280 ratios were obtained while using a Nanodrop 2000c spectrophotometer (Thermo Scientific, Paisley, UK). All of the RNA samples had rations of around 2.0. The integrity of the RNA isolated was tested while using an Experion automated electrophoresis system (Bio-Rad, Watford, UK). All of the samples in the study had RNA quality indexes (RQI) of >9.4. 100 ng of RNA from each sample was amplified and labeled with cyanine 3-CTP while using the one-color Low RNA Input Linear Amplification Kit according to the manufacturer’s instructions (Agilent, Stockport, UK). Labeled cRNA with a specific activity of at least 6.0 pmol Cy3 per μg cRNA were fragmented and hybridized on SurePrint G3 8× 60K Mouse gene expression microarrays (Agilent, Stockport, UK). Tab-delimited data files in text format that were obtained from the feature extraction were analysed using GeneSpring GX 12.0.1 software (Agilent, Stockport, UK) in order to compare the gene expression profiles between the samples. Data for a given gene were normalized to the median expression level of that gene across all samples. Fold change of 2 or more was used for significantly regulated genes using One-way ANOVA (*p*-value < 0.05). Pathway analysis from Pathvisio (http://www.pathvisio.org/)was used to set out the groups of differently expressed genes [52]. Hierarchical clustering was used to classify genes that were co-expressed across samples in groups according their functional relationship based on the fold change while using MeV software (http://www.tm4.org/mev.html) [53]. Gorilla carried out gene ontology (GO) analysis, which is the Gene Ontology enrichment analysis and visualization tool [54], available at http://cbl-gorilla.cs.technion.ac.il/.

### 4.5. Quantitative Real-Time Polymerase Chain Reaction Analysis

The cells were washed once with PBS and total RNA was extracted with the RNase Mini Kit following the manufacturer’s instructions, which included an on-column DNase treatment (QIAGEN, UK). For cDNA synthesis, 2 μg RNA was subjected to reverse transcription while using SuperScript III cDNA Synthesis system (Invitrogen, Paisley, UK), following the manufacturer’s manual with Oligo-dT as the primer. Real-time PCR (RT-qPCR) was performed while using SYBR select Master Mix chemistry (Applied Biosystems), following the manufacturer’s instructions. The sequence of primers that were used throughout this study were specifically designed to only bind the selected target using Gene Runner v3.1 Software (Hastings Software, Hastings, NY, US) and they were checked for specificity while using the National Centre for Biotechnology Information (NCBI) Primer-BLAST web tool [55]. Table 3 displays the sequence of primers. The thermal cycling and detection was carried out on a StepOne Plus real-time PCR system (Applied Biosystems, UK). The thermal cycle consisted of an initial uracil-DNA glycosylase activation of 2 min. at 50 °C to control PCR product carryover contamination, then a DNA polymerase activation of 2 min. at 95 °C, being followed by 40 cycles of 15 s at 95 °C, 60 s at 60 °C. A final extension of 5 min. at 72 °C followed and the reaction was stopped by extended incubation and cooling down to 4 °C. Melt curve analysis was conducted on the reactions to assay specificity. The expression levels of *Mmd* and *Csf2* mRNA transcripts were normalized to the housekeeping gene *QARS* while using the delta-delta Ct method [20].

### 4.6. Flow Cytometry Assay

Macrophages (1 × 10^6^) were harvested, passed through a nitex mesh to remove clumps, washed with PBS, incubated with Fc-Block (5 µg/mL αCD16/CD32, BD Bioscience, 1% mouse serum in RPMI) for 10 min. at room temperature, and the cell surface markers were then stained with conjugated antibodies for CD11b, CD115, MHCII, F4/80, or CD34 for 1 h at 4 °C. After several washing steps, the cells were resuspended in 200 mL PBS and subsequently run and analyzed on the FACS Canto flow cytometer (BD Bioscience) while using FACS Diva software and data analyzed using Kaluza software (Beckman Coulter, High Wycombe, UK). All of the values were normalized to their respective isotype control.

### 4.7. Proliferation Assay

#### 4.7.1. Cell Counting by Hematoxylin

Confluent macrophages were detached with cold PBS, seeded onto coverslips in 24-well plates (5000 cells/well) in 10% FCS-DMEM, and then allowed to attach for 24 h. Cells were starved in serum free media for 24 h and then stimulated for either 24, 48, 72 h, with 10 ng/mL of recombinant M-CSF. The cultures were washed with PBS and stained with hematoxylin. The number of cells was determined by counting in 10 random fields per each coverslip.

#### 4.7.2. BrdU Cell Proliferation Assay

The cells were harvested on the third day of the culture, seeded at a density of 4 × 10^4^ per well of 96-well plate and allowed to attach overnight in 10% FCS-DMEM. The next day, macrophages were treated with 10 ng/mL of M-CSF for 24, 48, 72 h, or left untreated as controls. Macrophage proliferation was assessed while using an anti-BrDU antibody detection assay kit, according to manufacturer’s instructions (Cell signalling, New England Biolabs, Hitchin, UK).

### 4.8. Western Blotting

The macrophages were lysed in SDS-PAGE sample buffer (63 mM Tris-HCL, pH 6.8, 2 mM Na_4_P_2_O_7_, 5 mM EDTA, 10% (*v*/*v*) glycerol, 2% (*w*/*v*) SDS, 50 mM DTT, 0.007% (*w*/*v*) bromophenol blue). The lysates were resolved by SDS-PAGE and transferred to nitrocellulose membranes. The membranes were blocked for non-specific binding for 2 h in 2% BSA (*w*/*v*), diluted in NATT buffer 50 mM Tris-HCl, 150 mM NaCl, 0.2% (*v*/*v*) Tween-20. The blots were then incubated overnight with 50 ng/mL primary antibody that was diluted in 0.2% BSA (*w*/*v*) in NATT buffer. The blots were washed with NATT buffer for 90 min. and incubated with HRP-conjugated secondary antibody (20 ng/mL in 0.2% BSA (*w*/*v*) diluted in NaTT buffer) for 2 h. After a further 90 minutes wash, the blots were subjected to ECL reagent and then exposed to Kodak X-ray film.

### 4.9. Data Analysis

Each figure represents one of at least three separate experiments, unless otherwise stated. The Western blots were scanned while using an Epson perfection 1640SU scanner using Adobe Photoshop 5.0.2 software. For gels, densitometry measurement was performed while using the Scion Image program. Data were expressed as mean ± SEM. Statistical analysis was performed while using GraphPad Prism (Version 5.0, GraphPad Software, San Diego, CA, USA). Statistically significant differences were determined using students *t*-test. P values equal or below 0.05 were considered to be significant.

## 5. Conclusions

We conduct a whole genome array in macrophages from wt and ko DUSP-4 deletion mice for the first time. The study reveals major effects on a number of genes, including those that are involved in macrophages differentiation. We go on to show that loss in MKP-2 has profound effects upon the proliferation and differentiation of macrophages. However, it is unclear whether these effects are directly linked to effects upon ERK signaling, but is related to the very early stage of macrophage formation. In addition, novel findings from this study highlight the significant role of MKP-2 in the immune response via regulating macrophage functions and adds new findings, which help in understanding the role of MKP-2 in immune biology.

## Figures and Tables

**Table 1:** Top 10 up-regulated Agilent (8× 60K) SurePrint G3 Mouse Gene Expression Array probe sets by LPS.

No.	FC-MKP-2^+/+^	FC-MKP-2^-/-^	Gene Symbol	Gene Name
1	4717.09	3292.84	*Il19*	interleukin 19
2	2975.03	1604.87	*Cxcl1*	chemokine (C-X-C motif) ligand 1
3	1850.59	1746.34	*Ptgs2*	prostaglandin-endoperoxide synthase 2
4	1758.98	1369.49	*Il1α*	interleukin 1 alpha
5	1574.15	663.96	*Gfi1*	growth factor independent 1
6	1094.3	5176.78	*Edn1*	endothelin 1
7	769.02	742.88	*Il6*	interleukin 6
8	710.97	446.13	*Cxcl2*	chemokine (C-X-C motif) ligand 2
9	434.82	425.43	*Dusp2*	dual specificity phosphatase 2
10	423.49	494.97	*Il1β*	interleukin 1 beta

A fold change (FC) of 2 or more was used for significantly regulated probes using one-way ANOVA (*p*-value < 0.05). Expression responses were analyzed in LPS treated cells and compared to that of untreated cells. The list is sorted according to WT macrophages.

**Table 2:** GO Analysis of the genes expressed between MKP-2^+/+^ and MKP-2^-/-^ macrophages with ≥2.0-fold change.

GO Term	Description	*p*-Value
GO:0009611	response to wounding	4.10E−14
GO:0050727	regulation of inflammatory response	1.97E−13
GO:0001817	regulation of cytokine production	2.75E−13
GO:0051239	regulation of multicellular organismal process	2.07E−12
GO:0032101	regulation of response to external stimulus	1.42E−11
GO:0048518	positive regulation of biological process	4.44E−11
GO:0023051	regulation of signalling	6.00E−11
GO:0010646	regulation of cell communication	8.51E−11
GO:0008284	positive regulation of cell proliferation	9.07E−11
GO:0050865	regulation of cell activation	1.63E−10
GO:0010941	regulation of cell death	1.32E−09
GO:0008009	chemokine activity	8.31E−08
GO:0032496	response to lipopolysaccharide	1.09E−08
GO:0001818	negative regulation of cytokine production	1.50E−08
GO:0045595	regulation of cell differentiation	7.88E−08
GO:0032680	regulation of tumor necrosis factor production	1.06E−07
GO:0042625	ATPase activity, coupled to transmembrane movement of ions	5.32E−05
GO:0017017	MAP kinase tyrosine/serine/threonine phosphatase activity	3.86E−04

*p*-value is the enrichment *p*-value computed according to the hypergeometric distribution model.

**Table 3:** Nucleotide sequences of the primers used for the analysis of gene expression by Quantitative Real Time Polymerase Chain Reaction amplification (qRT-PCR).

Primer Name	Sequence	PCR Product Size (bp)
*Qars*	Forward: 5′-GGACTCCAGCTGAGCGCTGCTC-3′ Reverse: 5′-GGTGGACTCCACAGCTTCCTCAAT-3′	138
*Csf-2*	Forward: 5′-TGCCTGTCACGTTGAATGAAGAGG-3′ Reverse: 5′-TGTCTGGTAGTAGCTGGCTGTCATGTTC-3′	164
*Mmd*	Forward: 5′-TGGCCGCTACAAACCAACGTG-3′ Reverse: 5′-CAAAGGCCCATCCCGTAGATCC-3′	156
*Edn1*	Forward: 5′- ACA CTC CCG AGC GCG TCG TA -3′ Reverse: 5′- TCT TGT CTT TTT GGT GAG CGC ACT G -3′	142

## Supplementary Materials

Supplementary materials can be found at https://www.mdpi.com/1422-0067/20/14/3434/s1.
