# Supplementary material for: Whole Genome Microarray Analysis of DUSP4-Deletion Reveals A Novel Role for MAP Kinase Phosphatase-2 (MKP-2) in Macrophage Gene Expression and Function
Source: Int J Mol Sci. 2019 Jul 12;20(14):3434. doi: 10.3390/ijms20143434 (PMC6679025; doi:10.3390/ijms20143434)
Supplement: Supplementary file 1 [file ijms-20-03434-s001.pdf]

International Journal of
Molecular Sciences

MDPI

Article

Whole Genome Microarray Analysis of DUSP4-Deletion Reveals A Novel Role for MAP Kinase Phosphatase-2 (MKP-2) in Macrophage Gene Expression and Function

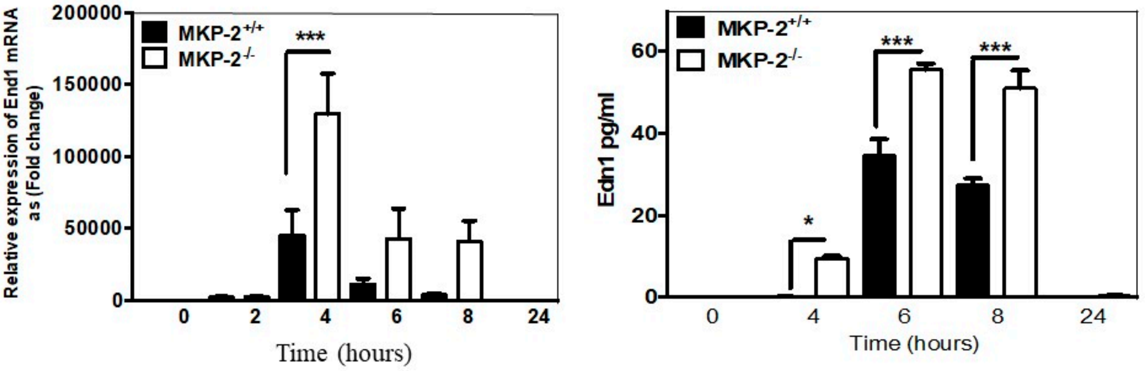

Supplementary Figure 1. MKP-2 deletion enhances LPS mediated Edn1 gene expression. (**A**) Macrophages were harvested, seeded into 6 -well plates (2 × 10^6) then stimulated with LPS (100 ng/mL) for the indicated times. Control cells were left untreated (0). Total RNA was prepared from cells. After reverse transcription, quantitative PCR analysis was performed on cDNA using primers designed to detect. Expression levels of Edn1 mRNA transcripts were normalized to the reference gene QARS using the delta-delta Ct method [20]. (**B**) Macrophages at a density of 2 × 10^6 were stimulated with LPS (100 ng/mL) for the indicated periods of time. Supernatant were collected and used immediately to assess Edn1 protein levels by ELISA. Error bars represent the mean ± SEM from five individual experiments. * $p < 0.05$, *** $p < 0.001$, Bonferroni's Multiple Comparison Test comparing MKP-2 $^{+/+}$ to MKP-2 $^{-/-}$.
